# Patient‐derived xenograft model in cancer: establishment and applications

**DOI:** 10.1002/mco2.70059

**Published:** 2025-01-19

**Authors:** Ao Gu, Jiatong Li, Meng‐Yao Li, Yingbin Liu

**Affiliations:** ^1^ Department of Biliary‐Pancreatic Surgery Renji Hospital Shanghai Jiao Tong University School of Medicine Shanghai China; ^2^ State Key Laboratory of Systems Medicine for Cancer Shanghai Cancer Institute Renji Hospital Shanghai Jiao Tong University School of Medicine Shanghai China

**Keywords:** cancer, multiomics, patient‐derived xenograft model, therapy, tumor progress

## Abstract

The patient‐derived xenograft (PDX) model is a crucial in vivo model extensively employed in cancer research that has been shown to maintain the genomic characteristics and pathological structure of patients across various subtypes, metastatic, and diverse treatment histories. Various treatment strategies utilized in PDX models can offer valuable insights into the mechanisms of tumor progression, drug resistance, and the development of novel therapies. This review provides a comprehensive overview of the establishment and applications of PDX models. We present an overview of the history and current status of PDX models, elucidate the diverse construction methodologies employed for different tumors, and conduct a comparative analysis to highlight the distinct advantages and limitations of this model in relation to other in vivo models. The applications are elucidated in the domain of comprehending the mechanisms underlying tumor development and cancer therapy, which highlights broad applications in the fields of chemotherapy, targeted therapy, delivery systems, combination therapy, antibody–drug conjugates and radiotherapy. Furthermore, the combination of the PDX model with multiomics and single‐cell analyses for cancer research has also been emphasized. The application of the PDX model in clinical treatment and personalized medicine is additionally emphasized.

## Graphical abstract

This review presents the establishment and application of patient‐derived xenograft (PDX) models, underscoring their extensive application in various therapeutic strategies including chemotherapy, targeted therapy, delivery systems, combination therapy, antibody–drug conjugates, and radiotherapy. Additionally, the integration of PDX models with multiomics and single‐cell analysis in cancer research is highlighted. Pertinent clinical studies are further examined and discussed, thereby advancing the development of cancer treatment strategies.

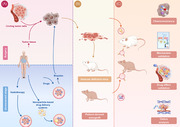

## 1 INTRODUCTION

Cancer is a highly fatal disease and has emerged as one of the foremost contributors to global mortality. The utilization of in vitro and in vivo experiments is indispensable in oncology research, particularly with respect to employing in vivo models that offer a more comprehensive understanding of tumor behavior and feedback on treatment efficacy.^1^ The choice of an appropriate in vivo model is critical and should be guided by the specific objectives of the experiment. The currently utilized mouse models primarily include chemical carcinogen‐induced tumor mouse models, genetically modified mouse tumor models, allograft mouse tumor models, and xenograft tumor models.^2^

To comprehensively capture human tumor heterogeneity and effectively regulate specific genes, one of xenograft tumor models, patient‐derived xenografts (PDX) model offer enhanced reliability for studying and validating therapeutic strategies, intratumor cell–cell communications, and omics analysis.^3^, ^4^ PDX models offer a promising approach to enhancing clinical decision‐making by preserving essential characteristics of the patient's tumor tissue, including histopathological structure, genomic features, tumor heterogeneity, drug responsiveness, and chromosomal instability.^5^ In contrast, models utilizing patient‐derived samples are considered the most practical and available option for accurate representation.^6^, ^7^ Despite the inherent limitations of current animal models in faithfully replicating intricate microenvironments and capturing tumor heterogeneity, PDXs have emerged as promising alternatives.^8^ By implanting small tumor fragments obtained through surgical dissection from cancer patients into highly immunodeficient mice, PDX models facilitate efficient tumor growth and subsequent transplantation into secondary recipient mice. The PDX models are considered suitable for conducting experiments at the P2 generation.^9^ PDX mouse models have been employed to replicate tumor heterogeneity and predict clinical outcomes, thereby evaluating and validating the efficacy of antitumor compounds.^10^

Moreover, the utilization of PDX models presents novel prospects for precision medicine and personalized healthcare.^11^ Precision medicine holds the potential to transform cancer care, as increasing evidence indicates that patients receiving personalized therapy exhibit improved clinical outcomes.^12^ The identification of mutations in individual genetic profiles during treatment and their correlation with drug sensitivity is crucial for the precise management of advanced cancer patients.^13^ To fully realize the potential of precision medicine, it is imperative to develop models that comprehensively capture the molecular heterogeneity of tumors.

**FIGURE 1:**
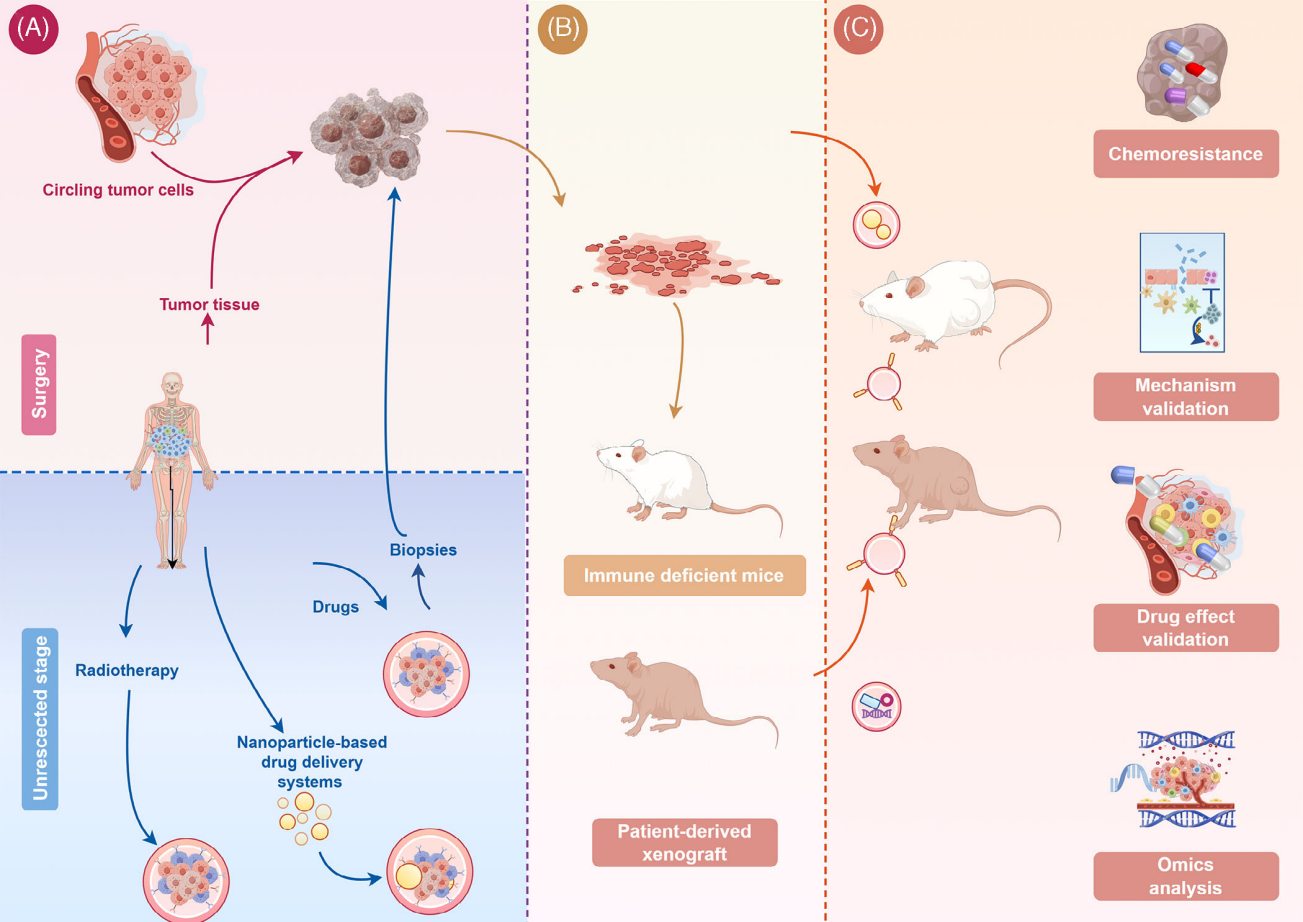
Patient‐derived models: tumor cell resource, establishment, and application of PDX models. (A) PDX models can be derived from resected tumor, circulating tumor cell (CTC) from the blood and biopsy. (B) PDX are established based on the immune deficient mice. (C) PDX models can be utilized on the investigation of the chemoresistance, mechanism validation, drug effect validation, and omics analysis.

This review extensively covers the establishment process, advantages, and limitations of PDX models, emphasizing current technologies for enhancing PDX model development. Additionally, this study highlights the pivotal role of PDX models in exploring mechanisms, chemotherapy regimens, targeted therapies, immunotherapies, combination therapies, and other innovative cancer treatment approaches. PDX models offer crucial solutions to various challenges, including assessing new drug effectiveness, identifying drug‐sensitive and drug‐resistant targets, and elucidating the mechanisms of drug resistance. The integration of single‐cell analysis and multiomics techniques has also been introduced to elucidate therapeutic strategies for cancer (Figure 1). In the subsequent discussion, we systematically elaborate on the construction and application of PDX models in the context of various cancers. Additionally, to enhance readers’ comprehension, we provide a detailed explanation of the use of lung cancer as illustrative examples because it is the most prevalent form of cancer worldwide.

## 2 ESTABLISHMENT OF THE PDX MODELS

A deeper understanding of the research status and methods used to construct PDX models is crucial. In this section, we provide a systematic overview of the historical development and current state of PDX models, elucidate specific techniques for their establishment, introduce efficacy evaluation criteria, analyze tumor sources for model construction, and discuss limitations associated with this model. Furthermore, to broaden our discussion scope, we initially explained the construction process of patient‐derived organoid‐based xenograft (PDOX) models and compared them with PDX models.

### 2.1 History and current status of PDX models

The high demand for PDX models stems from their ability to effectively preserve the molecular characteristics and heterogeneity of tumor tissue. As early as 200 years ago, scientists attempted to cultivate various types of human tumor tissues in diverse animal models. However, owing to the lack of theoretical guidance and equipment support, the majority of these research endeavors ended in failure. The earliest documented instance of a PDX model, representing a pioneering effort, dates back to 1969 when Rygaard and Povlsen extracted colon adenocarcinoma tissue from a patient and subsequently implanted the tumor fragments into immunodeficient nude mice.^14^ The time required for model establishment, the success rate of tumor engraftment, the range of applicable tumors, and the cost of establishing a mouse model are all critical factors that must be considered in preclinical studies. They have been established for various cancer types, such as breast,^15^ gastric,^16^ colorectal,^17^ glioma,^18^ and head and neck malignancies.^19^ Tumor tissues can be engrafted heterotopically in diverse sites, including the intracapsular fat pad,^20^, ^21^ the anterior eye compartment,^22^ under the renal capsule,^23^ subcutaneously, and orthotopically at the cancer sites.^24^ To prevent rejection in mouse models, conventional PDX models typically use immunocompromised strains such as athymic nude mice, severe combined immunodeficiency (SCID) mice, nonobese diabetic/SCID (NOD–SCID) mice, NOD–SCID/IL2λ‐receptor null (NSG) mice, BALB/cRag2 null/IL2λ‐receptor null mice, and Rag‐2 null/Jak3 null mice. Nude mice have been acknowledged as standard recipients for generating PDX models because of their substantial efficacy and cost effectiveness.^25^ However, the presence of an activated innate immune system and T‐cell leakage as these cells age in nude mice limits the feasibility of human cancer transplantation. NSG strains, which represent the most immunocompromised models available and exhibit the highest potential for engraftment efficiency, significantly increase the success rate of engraftment.

Different tumor types vary in the time required for successful PDX establishment, ranging from days to months (Table 1). The established PDXs presented a significantly shorter time to passage in subsequent generations, indicating increased aggressiveness. The duration required for tumor engraftment varies depending on the type of tumor, the site of implantation, and the recipient strain. Typically, this period ranges from 2 to 4 months; however, it is recommended to refrain from concluding engraftment failure until at least 6 months have elapsed. The establishment of the first gastrointestinal passage was documented to occur within a range of 1–4 months, with an average duration of 2.6 months. Following serial passage transplantation, the duration of tumor growth stabilizes, with pancreatic cancer and gallbladder cancer typically requiring about 1–2 weeks to reach a specific tumor.

**TABLE 1:** PDX successful rates of various tumor were listed over the past 3 years.

Tumor type	Successful rate	Tumor cell source	Time (median)	Mouse strain	Implantation site	References
Colorectal Esophageal Gastric/gastroesophageal junction Breast Gallbladder Cholangiocarcinoma Small bowel cancer	19/36 (52.7%)	Biopsies Resected tissue	111 days	NSG	Subcutaneous	DiPeri et al.^40^
Cholangiocarcinoma	19/49 (38.8%)	Biopsy Primary tumors Metastasis	4.7 months	NOD/SCID	Subcutaneous	Serra‐Camprubí et al.^41^
Prostate cancer	13/63 (20.6%) primary 28/145 (19.3%) metastasis	Resected primary Metastasis	About 2 months	NOD–SCID	Renal capsule	Risbridger et al.^42^
Endometrial cancer	13/32 (40.6%)	Resected primary tissue		NSG	Subcutaneously	Bonazzi et al.^43^
Triple negative breast cancer	62/269 (23%)	biopsy		NOD–SCID	Orthotopic	Echeverria et al.^44^
Non‐small cell lung cancer	50%	Resected primary tissue	85 days (37–440 days)	NSG	Subcutaneous	Hynds et al.^45^

The engraftment rate of patients with colorectal cancer (CRC)^26^ was greater than that of patients with esophageal cancer, whereas patients with gastric cancer presented a lower engraftment rate. CRC PDX models are relatively straightforward to establish, with rates exceeding 75%.^27^, ^28^, ^29^, ^30^, ^31^, ^32^, ^33^ Melanoma has a success rate of 28% in SCID mice, whereas the success range for head and neck cancer (HNSCC) biopsies in mice is between 29 and 44%.^34^ Pancreatic cancer also has a relatively high engraftment rate in patients with digestive system diseases. The success rate of constructing lung cancer and prostate cancer PDX models is relatively low. To address this issue, researchers have implanted fresh patient‐derived tumors in the subrenal capsule of NOD/SCID mice, leveraging the area's enhanced blood flow for quicker tumor microvasculature development. This method achieved over 95% engraftment in a cohort of 14 diverse lung cancer PDX samples and over 90% efficient engraftment.^35^, ^36^ The abundance of evidence further supports the notion that PDX models derived from individuals with advanced‐stage disease and poorer prognoses exhibit notably higher engraftment rates.^37^, ^38^, ^39^

### 2.2 How to construct PDX models

**FIGURE 2:**
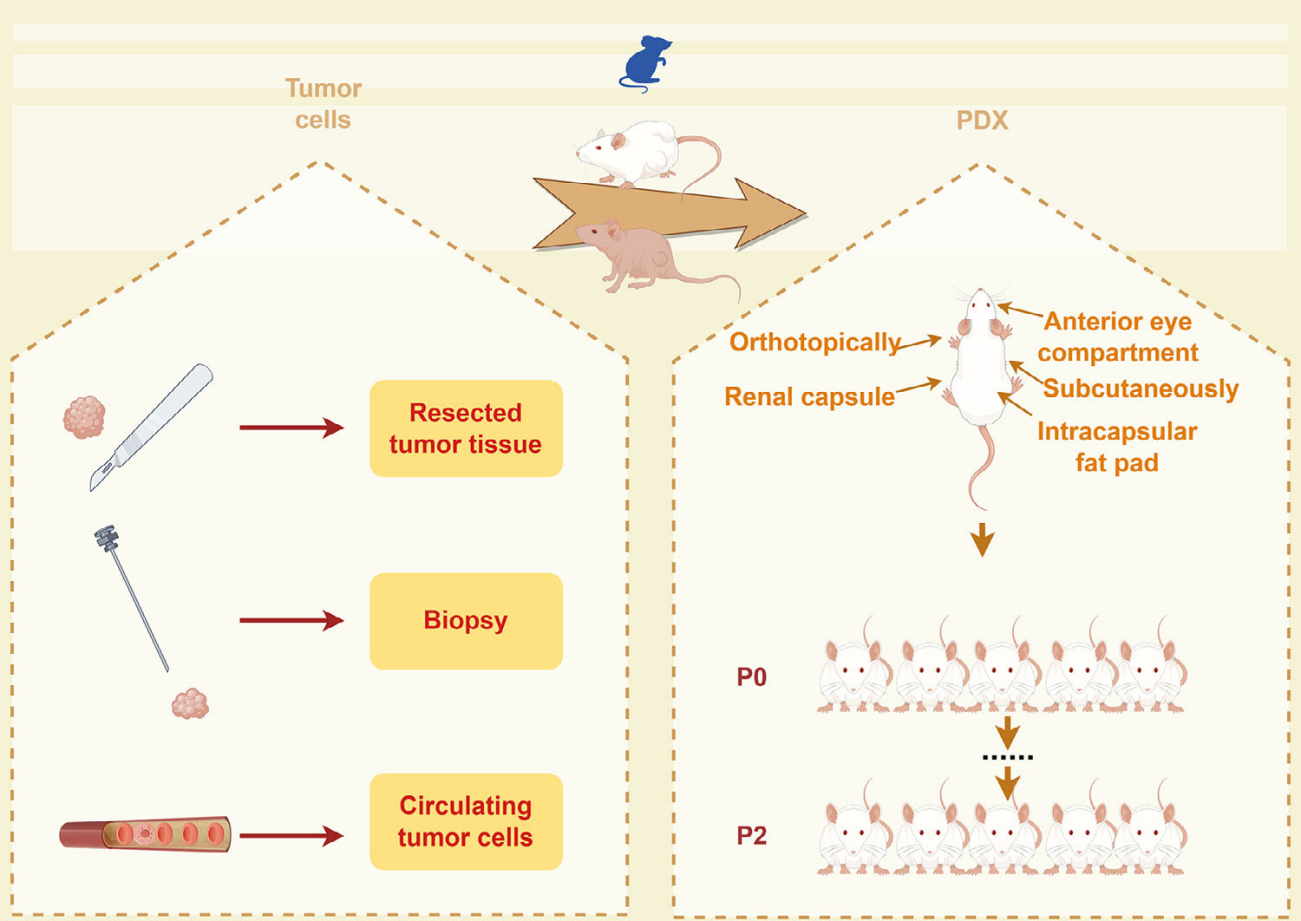
PDX models are established from the implanted tumor cells into the different sites of mice. Tumor cells can be derived from resected tissues, biopsy, CTCs. The implanted sites of immune deficient mice include intracapsular fat pad, the anterior eye compartment, under the renal capsule, subcutaneously, and orthotopically at the cancer site. The PDX models are considered suitable for conducting experiments at the P2 generation.

PDX models preserve original tumor histology, molecular features, and genetic alterations, offering advantages over conventional cell line‐derived xenografts and other models.^46^ The following process can generally be used to generate PDX models for the majority of solid tumors (Figure 2). Tumor tissue transplantation involves sectioning into 3–5 mm^3^ fragments, employing abundant tumor material, or using minced tumors. Before transplantation, the tumor samples were washed three times with cold phosphate‐buffered saline (PBS) and penicillin/streptomycin. An appropriate engraftment site is subsequently selected. Tumor fragment‐derived PDX models have also been reported to undergo dissociation and enzymatic digestion with collagenase to generate single‐cell suspensions for further establishment.^47^ For the establishment of hematological malignancies, samples were intravenously injected into NSG mice at 10^5^–10^6^ viable cells per mouse.^48^ Hematological malignancy can be recognized through the identification of a significant proportion of human leukemia cells in the peripheral blood of mice, as well as the infiltration of malignant cells into the bone marrow and spleen, utilizing flow cytometry for analysis.^49^ Among the chemotherapy‐treated and nonchemotherapy groups, varying rates of successful transplantation were observed, with some demonstrating lower rates and others showing higher rates. The success of transplantation may be associated with the acquisition of chemoresistance resulting from the therapeutic regimen.^50^, ^51^ A clinically relevant glioblastoma (GBM) model was established via human GBM biopsies. In contrast to conventional PDX models in which tumor tissue is directly implanted into mice postsurgery, these biopsies are sectioned into 0.3 mm^3^ samples, cultured in agar to generate spheroids over a period of 18 days, and subsequently engrafted into the brains of athymic nude rats. This methodology exhibited an impressive success rate of 96% (28 out of 29 tumors) and faithfully recapitulated the disease progression observed in humans.^52^ With respect to breast cancer (23% success rate),^44^ triple‐negative tumors exhibit the most rapid growth, which aligns with the aggressive clinical presentation of this disease. The rate of engraftment emerged as a prognostic indicator for patient survival, even among those with newly diagnosed disease who did not present with detectable metastases at the time of surgery.^53^ Accurate identification and characterization of tumor molecular changes and genetics are crucial for screening targeted drugs and treatments.

### 2.3 Therapy effect evaluation criteria

Therapeutic efficacy was evaluated by comparing the initial tumor volume change at that time to its baseline value. The best response was the minimum value of tumor volume change for more than 10 days. The average tumor volume change from the initial day to the last day was also calculated. The best average response (BestAvgResponse) was defined as the minimum value of this average for more than 10 days. The criteria for tumor response (mRECIST) followed the RECIST criteria^54^ and were as follows: complete responses, BestResponse < −95% and BestAvgResponse < −40%; partial responses, BestResponse < −50% and BestAvgResponse < −20%; partial responses, BestResponse < 35% and BestAvgResponse < 30%; progressive diseases, not otherwise categorized.^55^ Based on the growth patterns of PDXs, certain cases established predefined thresholds to categorize responses: responders exhibited less than 100% growth, partial responders demonstrated 100–300% growth and a volume that was ≤50% of the matched vehicle, and nonresponders showed greater than 300% growth. These classifications were determined by evaluating the change in graft volume from treatment initiation. Notably, during the treatment period, the tumor volume reached the maximum ethical limit of 1000 mm^3^, necessitating earlier tumor harvesting to comply with animal ethics approval.^42^

### 2.4 Model established tumor source

A significant challenge in small cell lung cancer (SCLC) cancer biology research is the limited availability of tumor tissue, prompting the creation of PDXs from transbronchoscopic biopsy specimens.^56^, ^57^ Established models indicate that pERK, a critical component of the MAPK pathway, serves as a potential biomarker for predicting chemorefractory status before chemotherapy initiation, identified using a targeted next‐generation sequencing gene panel based on PDXs. Norton et al.^58^ integrated CRISPR/Cas9 technology with PDX models derived from CTCs or tumor biopsies, identifying neddylation as a crucial regulator of the neuroendocrine cell state and a promising therapeutic target for small cell carcinomas. In clinical practice, insufficient availability of tumor tissue sometimes necessitates alternative methods such as biopsy and CTCs to obtain additional tumor samples for establishing PDX models. Non‐small cell lung cancer (NSCLC) tumor tissues were implanted in mice to create ptPDX models, from which CTCs were isolated and used to generate CTC derived xenograft tumor models. Subsequent single‐cell RNA sequencing and validation elucidated potential mechanisms of chemoresistance in human NSCLC PDX models.^59^

### 2.5 Establishment of PDOX models

Patient‐derived organoids (PDOs) are patient tumors established in vitro and are derived from either adult stem or pluripotent cells.^60^ Although 3D organoids can partially recapitulate certain epithelial cell features in the body, the absence of crucial environmental factors, such as stromal cells, immune cells, and the vascular system, precludes faithful reproduction of the intricate internal microenvironment.^61^ The introduction of PDOXs represents a significant advancement in the field, as it allows for direct injection of organoids into mice, thereby overcoming the limitations associated with in vitro PDO models. By combining the advantages of both PDO and PDX, the success rates have significantly increased, enabling rapid establishment of PDOX under specific environmental conditions. Moreover, with the aid of matrix support, xenografting into mice can be more reliable. However, it is evident that there are certain shortcomings to address; namely, the culture process of PDO faces passive selection for tumor cell subtypes, and subsequent xenografting involves another environment‐dependent selection process. Additionally, achieving successful engraftment when PDOs are injected into PDX models remains a challenge.

The difference of the PDX and PDOX models highlighted that the PDX model was established using patient tumor tissue, which consists of various cell components, including immune cells and cancer‐associated fibroblasts (CAFs). While immune cells infiltrating the resected tumor may persist for one or two passages, CAFs can exist for a longer duration. CAFs in PDX models are composed primarily of human‐derived CAFs and express gene signatures associated with patients' tumors. On the other hand, PDOX models rely on stable‐passage PDOs, which are predominantly composed of epithelial cells, and exhibit an increased presence of murine CAFs. This may impede the future application of PDOX in investigating tumor–CAF communication.

### 2.6 Limitations of PDX models

**FIGURE 3:**
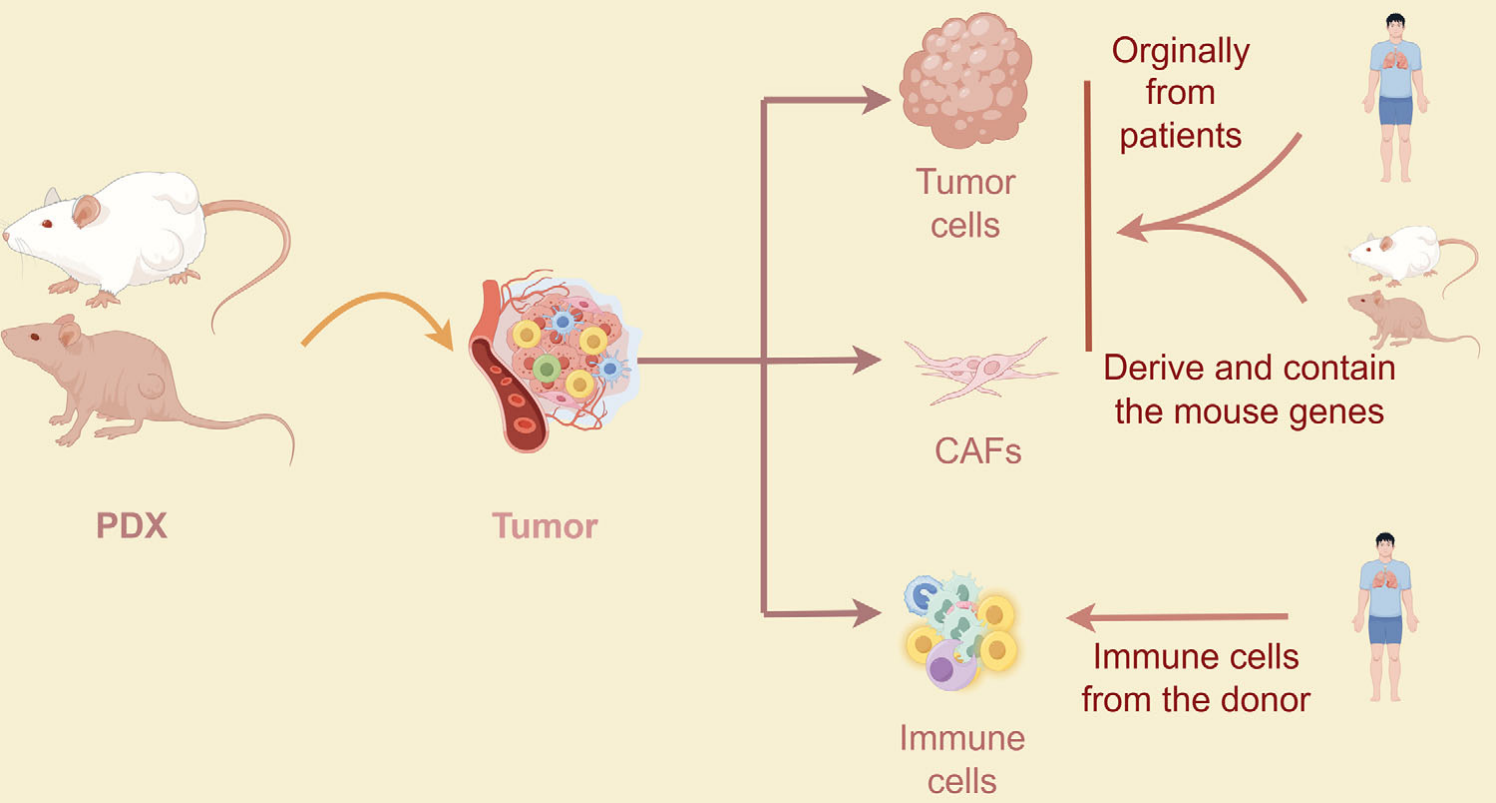
Tumor tissue derived from PDX comprises tumor cells, CAFs, and immune cells, thereby recapitulating the tumor microenvironment. The PDX tumor tissue harbors a combination of genes originating from both patients and mice within its constituent tumor cells and CAFs. Immune cells, including peripheral blood mononuclear cells (PBMCs) and CD34+ cells, are typically sourced from donors to establish a humanized immune system in mouse models.

Although existing PDX models have shown commendable efficacy, there are still aspects that require further refinement. Given the intricate nature of the tumor microenvironment, preserving intercellular interactions and TME structural components is paramount in investigating clinical tumor tissue intercell signal transduction and holds significant implications for studying drug response (Figure 3). Research has demonstrated that PDX models preserve key characteristics, including tissue architecture and biological behaviors.^62^ However, murine stromal cells and murine‐derived chemokines and cytokines (such as IL‐2 on human T cells and IL‐15 on NK cells) replace human stromal cells to facilitate immune cell activation during the second establishment.^63^ Human stromal cells gradually give way to their murine counterparts upon transplantation into immunodeficient mice, indicating that implanted human cancer cells recruit murine stromal cells to their microenvironment over time.^64^ Notably, differences exist between human and murine stromal cells, particularly in the sensitivity of cytokine secretion. For example, human IL‐2 effectively stimulates murine T‐cell proliferation, whereas mouse‐derived IL‐2 shows reduced efficacy in activating human T cells.^65^ IL‐15 binds to both human and mouse IL‐15 receptor α with similar affinities, suggesting that IL‐15 produced by PDXs may affect murine stromal cells.^66^ To address species‐specific concerns, coimplantation of human CAFs and tumor cell suspensions from PDXs into secondary recipient mice offers an ideal platform for studying interactions between human tumor cells and stromal cells.^64^

The role of the immune system in tumor progression and treatment highlights the critical need for PDX models in evaluating cancer immunotherapies during preclinical stages.^67^ PDXs are typically established in immunodeficient mice, such as NSG mice lacking T, B, and NK cells, which compromises their innate immune function. This conventional approach limits understanding of the dynamic interactions between tumors and the immune system. However, some lung cancer PDX models in early passages have shown patient‐derived immune cell coexistence.^68^ To effectively assess immunotherapy efficacy, human immune components must be introduced into humanized mice. These mice are engineered by transplanting total peripheral blood, particularly lymphocytes, from healthy donors or patients, though this approach often triggers significant graft‐versus‐host disease (GvHD) within 2–5 weeks, limiting study duration and translational relevance. Su et al.^69^ provide an alternative method of posttransplant treatment with AMD3100 to significantly alleviate GvDH. Alternatively, HIS mice are created by engrafting CD34‐positive human hematopoietic stem cells from sources like umbilical cord blood or bone marrow into immunodeficient mice, sometimes in combination with other human immune tissues (such as bone ossicles or human thymic tissue). HIS mice can also be generated by irradiation followed by CD34+ Hematopoietic stem/progenitor cells (HSPC) engraftment.^70^, ^71^ PDX tumors in mice engrafted with autologous CD34+ HSPCs exhibit infiltration of patient immune cells, including innate (e.g., macrophages) and adaptive (e.g., T cells) immune subtypes. Transcriptional analysis reveals activated and exhausted T cells within the tumor microenvironment, while macrophage signatures suggest a protumor role by promoting VEGF‐A production, crucial for PDX tumor progression in experimental settings.^72^

In addition to the long time needed to establish and high cost, the properties of the original cells cannot be obtained from the tissues of PDX models. However, studies have demonstrated that PDX underrepresent subclonal heterogeneity, a factor that may facilitate further application, although most clonal mutations are preserved. This underrepresentation may be attributed to sampling bias resulting from spatial heterogeneity, differential capacities for engraftment and proliferation postinjection, or tumor evolution and selection during PDX passages. Hoge et al.^73^ performed a comparative analysis of primary tumors (PTs) and their corresponding PDXs across multiple passages and revealed that, compared with earlier‐passage PDXs, later‐passage PDXs exhibit significantly reduced genomic similarity to their parental PTs, indicating genomic divergence. There is an average of 10–20% copy number (CN) between PTs and PDXs, indicating continuous diversification of CN landscapes during model propagation. This diversification is likely attributable to a combination of subclonal dynamics and ongoing genomic instability.^73^

The comprehensive investigation of the PDX model presents significant prospects for the advancement of pharmaceutical and clinical medicine. While PDX models offer promising advancements in cancer research, it is essential to acknowledge and tackle their significant limitations to make them more accessible for translational and preclinical studies. Using PDX models requires substantial financial investment and access to well‐equipped laboratory facilities. Key challenges that need attention and standardization to promote wider adoption include the time needed to generate PDX tumor tissues, the substitution of human stroma with murine stroma, and the current lack of immune system assessment.

PDX models are not suitable for early‐stage cancers due to their low success rate and the prolonged tumor latency of 4–6 months, which is a significant limitation for personalized medicine. When creating metastatic models, both heterotopic and orthotopic grafting methods, as well as injecting patient‐derived tumor cells into mice via the tail vein, each have their inherent advantages and limitations. Spontaneous metastasis in grafted tumors occurs slowly with a low rate, whereas injecting tumor cells results in a higher metastatic rate but presents challenges related to unrealistic heterogeneity, often leading to most cells being confined to the lungs rather than spreading to other organs.^74^

## 3 APPLICATIONS OF PDX MODELS

An optimal preclinical model should comprehensively replicate and preserve the full spectrum of characteristics inherent to the patients’ tumor. The PDX models faithfully recapitulate the parental tumor characteristics and accurately simulate authentic tumor–stromal cells interactions, thereby effectively reproducing drug responses and describing the subclonal heterogeneity of the tumor landscape. This would significantly enhance our comprehension of tumor evolution and function as a crucial instrument for precise pharmacological investigations.

Besides preserving the in vivo structure, PDX models can amplify original cancer cells and enhance the mutation profile of cancer genomes.^64^ These models maintain genetic profiles and intratumor heterogeneity, making them invaluable tools for studying tumor progression mechanisms, refining drug treatment strategies, and exploring cell–cell communication.^75^

**FIGURE 4:**
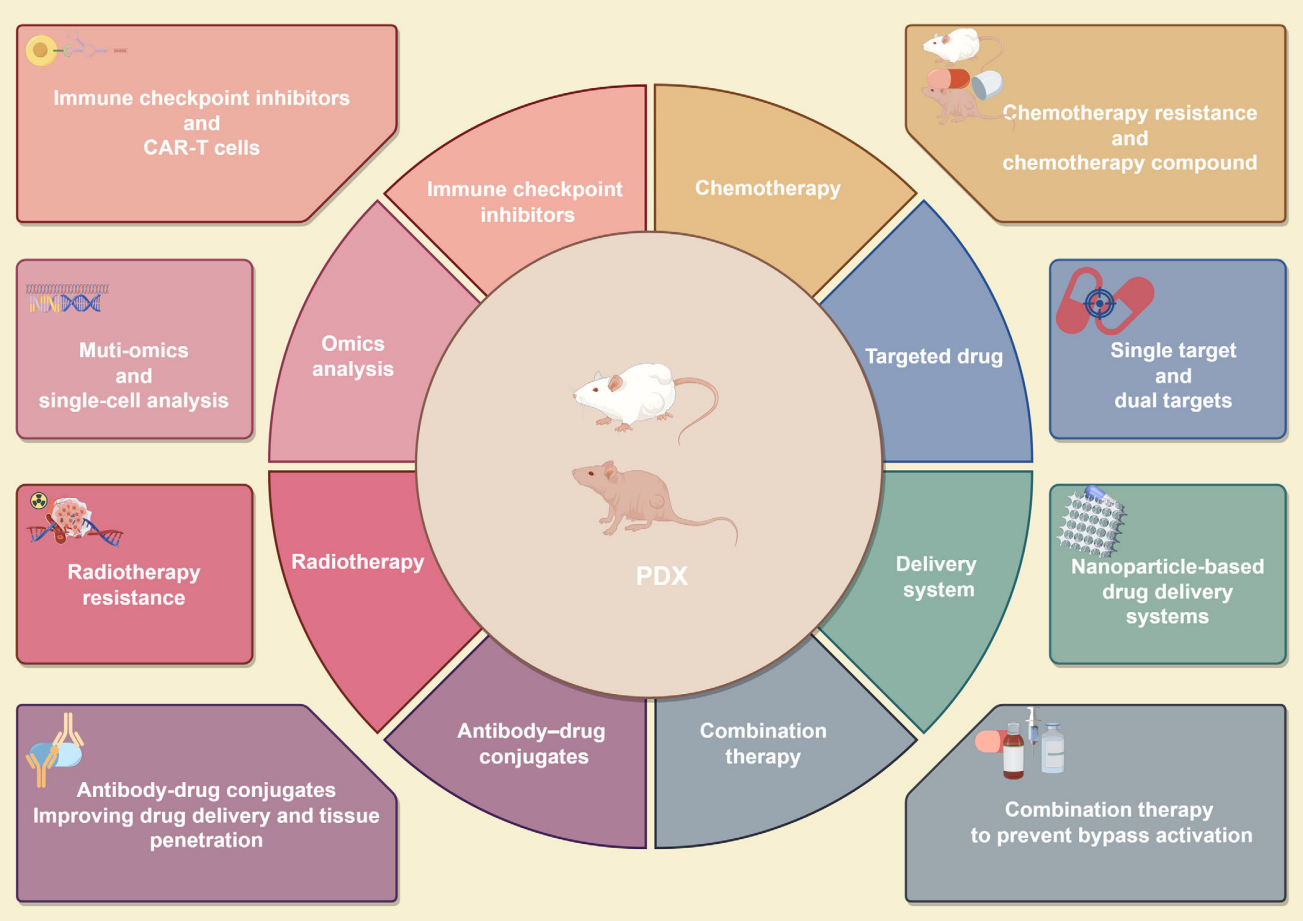
Applications of PDX models promote the investigation of drug validation and omics analysis. The utilization of PDX models facilitates drug validation and omics analysis, providing a clinically relevant platform for investigating drug treatment strategies, resistance mechanisms, and conducting in‐depth multiomics analyses.

In this section, we elucidate the application of the PDX model in the investigation of tumor progression mechanisms and the exploration of therapeutic strategies (Figure 4). Specifically, we highlight its utilization in the development of immune checkpoint inhibitors, chemotherapy drug research, targeted drug discovery, nano drug delivery system (DDS) development, combination therapy research, antibody–drug conjugate (ADC) exploration, and radiotherapy research, among other areas. Furthermore, we discuss advanced findings that integrate the PDX model with omics technologies, including multiomics studies and single‐cell analysis. Notably, we emphasize the clinical research outcomes associated with this model to augment the clinical relevance of this paper.

Notably, the application of PDX models spans a broad spectrum of tumor types, which makes exhaustive enumeration and detailed discussion of all instances difficult. Given the high incidence and mortality rates associated with lung cancer (LC), this section specifically highlights the utilization of PDX models for this type of tumor. Moreover, we address significant advancements in the application of PDX models in the context of pan‐cancer.

### 3.1 PDX models in investigation of the tumor progression mechanism

PDX models retain genetic profiles and intratumor heterogeneity, making them valuable for studying variations across different regions within primary lesions and metastases. They contribute significantly to understanding chemoresistance, addressing a current lack of well‐defined laboratory models that accurately reflect clinical cross‐resistance, which hinders translational research.^76^ Unlike genetically engineered mouse models lacking clinical relevance or cell lines showing comparable chemosensitivity pre‐ or posttreatment, PDX models faithfully replicate patient responses to DNA‐damaging treatments. For instance, PDX models treated with first‐line EP or EC and olaparib plus TMZ (OT) accurately replicated patient reactions. Genomic and transcriptional analyses of relapsed cross‐resistant SCLC PDX models revealed recurrent MYC paralog amplifications on ecDNA on relapsed cross‐resistant SCLC PDX models, highlighting their utility in studying treatment responses. Acquired chemo‐resistant acute lymphoblastic leukemia PDX models were established, and combined with CRISPR/Cas9 screening revealed the multiple resistance‐related genomic mutations.^77^

Zhang et al.^78^ utilized PDX models to highlight the pivotal role of ADCY10 in downstream targeting, influencing ferroptosis and offering potential for ferroptosis‐based therapies. Cai et al.^79^ demonstrated in an NSCLC PDX model that ERK inhibitors can induce epithelial–mesenchymal transition (EMT) in cells with abnormal MAPK signaling, suggesting a mechanism for tumor relapse in NSCLC following ERK inhibition. Noronha et al.^80^ found that TKIs activate the GAS6–AXL pathway in human lung cancer cells, promoting the survival of persister cells similar to the SOS pathway response.

As tumor samples originate from clinical patients, controlling specific gene mutations in the original tissues used for PDX engraftment is challenging. Gene expression changes are inferred from clinical pathology findings. Certain gene mutations were grouped based on pathology results, guiding subsequent treatments.^81^, ^82^, ^83^ Western blot analysis can reveal varying levels of specific expression in collected samples.^84^ Liu et al.^85^ demonstrated with PDX models that integrating multiomics subtyping and their characteristics aids in selecting treatments for SCLC effectively. Zhan et al.^86^ observed increased HOXB13 and EZH2 expression in cisplatin‐sensitive PDX samples posttreatment, suggesting cisplatin induces their upregulation. They identified a new network involving cisplatin/HOXB13‐ABCG1/EZH2/Slug, offering insights into lung adenocarcinoma metastasis and resistance to cisplatin therapy, essential for assessing treatment efficacy and predicting patient responses.^86^

Cell lines derived from PDX tumors, where specific genes are manipulated to alter their expression levels, significantly contribute to a comprehensive understanding of chemoresistance mechanisms. Establishing MYCN‐overexpressing PDX models has highlighted MYCN's crucial role in driving chemoresistance in SCLC.^87^ PDX tumors from NSG mice were dissociated into tumor cells and then ex vivo infected with lentivirus carrying MYCN cDNA. Following a 16‐h incubation period postextraction and lentivirus introduction, the modified cells were reintroduced into the flanks of NSG mice. Subsequent validation confirmed that MYC overexpression in two highly chemo‐sensitive SCLC PDX models induced complete chemoresistance, emphasizing the significant impact of these oncogenes on chemoresistance development in SCLC.

### 3.2 Exploration treatment strategies as preclinical models

There is growing evidence supporting the potential of PDX models to accurately predict the efficacy of both traditional and innovative anticancer treatments, highlighting their promise in conducting “coclinical trials.” This approach enables concurrent in vivo and clinical investigations to identify effective therapeutic targets.

While advancements have enhanced the functional testing of live patient tumor cells, drug screening technologies remain limited by the requirement for substantial cell quantities. One effective strategy to tackle these challenges involves directly testing drug compounds on patient samples. Consequently, large‐scale drug screening of patient tumors has increasingly focused on patient‐derived models, including PDOs, cell lines, and PDXs, which offer abundant malignant cells. This section explores the role of PDXs in cancer treatment, including immunotherapy, chemotherapy, targeted therapy, drug delivery systems (DDSs), combination therapy, ADCs, and radiotherapy.

#### 3.2.1 Immune checkpoint inhibitors investigations

The immune system plays a crucial role in regulating and eliminating cancer.^88^ However, within the context of malignancy, various mechanisms of immune suppression can hinder the ability to mount an effective antitumor immune response.^89^ Investigations on immune checkpoint inhibitors typically revolve around the introduction of the immune system, with a focus on studying infiltrated immune cells in PDX tissue or humanized mice. Therefore, enhancing the establishment of the immune system in PDX models will be a crucial area of emphasis in future research to enhance the accuracy and reliability of our findings.

The use of immune checkpoint inhibitors, particularly antiprogrammed death 1 (PD1)/PD‐ligand 1 (PD‐L1) agents, has markedly improved disease control in NSCLC, which represents about 90% of all lung cancer cases.^90^ SCLC, with its increased mutation frequency, suggests potential immunogenicity and responsiveness to immune checkpoint inhibitors.^91^ The effectiveness of immunotherapy (IOT) in NSCLC is significantly influenced by PD‐L1 levels, along with variations in sex, histology, and concurrent treatments.^92^ However, the impact of sex on responses to IOT remains contentious, especially in the context of cancer. The response of infiltrating immune cells to PD‐L1 blockade treatment was investigated in gastric cancer PDX models, revealing that tissue‐resident memory T cells play a crucial role in the antitumor response.^93^ The efficacy of a supramolecular peptide in optimizing ICI therapy was confirmed via PBMC‐injection PDX models of hepatocellular carcinoma,^94^ which demonstrated that the PBMC injection method enhances the investigative scope of drug effects.

Anoblie et al.^95^ focused on investigating the impact of sex hormones and identified estrogen receptor α (ERα) as a significant predictor of pembrolizumab response, independent of sex and PD‐L1 levels. They also noted a direct association between ERα expression and PD‐L1 expression, particularly in female patients.^95^ Using a humanized NSG PDX model, they engrafted female mice with human hematopoietic CD34^+^ cells from a single donor, ensuring similar levels of circulating monocyte‐ and lymphocyte‐derived lineages initially. Their study highlights the potential of 17‐β‐estradiol/ERα status to predict pembrolizumab efficacy in NSCLC patients.

The intricate immune‐suppressive milieu within solid tumors, coupled with the absence of specific tumor targets, presents challenges in effectively deploying chimeric antigen receptor T (CAR‐T) cell therapy.^96^ While CD19‐CAR‐T cells have proven highly effective in B‐cell‐derived hematological malignancies, their application in solid tumors has shown limited efficacy.^97^ Cao et al.^98^ investigated the impact of AXL‐specific CAR‐T cells in NSCLC. Additionally, combining microwave ablation (MWA) with AXL–CAR‐T cells demonstrated a synergistic effect, boosting T‐cell cytotoxicity and hindering distant metastasis.

Based on the above studies, the PDX model offers a valuable platform for investigating the effects of immunotherapy in a more clinically relevant context. While current studies have produced promising outcomes, several challenges remain to be addressed. These include strategies to mitigate or prevent immune‐related side effects, methods to increase therapeutic efficacy, and approaches to optimize the selection of appropriate treatment regimens for individual patients using PDX models. Future research on immune checkpoints utilizing PDX models can focus on several key areas: first, the development of more precise and personalized treatment strategies tailored to the specific needs of individual patients; second, an in‐depth investigation into combination strategies of immunotherapy with other treatment modalities to optimize therapeutic outcomes; third, an emphasis on the long‐term effects and safety profiles of immunotherapy to ensure its clinical reliability; and, finally, the exploration of novel targets and signaling pathways to broaden the scope of immunotherapy applications.

#### 3.2.2 Chemotherapy investigations

Chemotherapy is a widely accepted treatment for combating tumors and has been thoroughly validated across various types of cancer.^99^, ^100^, ^101^, ^102^, ^103^ Despite its efficacy, the emergence of chemotherapy resistance remains a major contributor to cancer‐related deaths worldwide.^104^ Furthermore, there is a distinct lack of reliable methods for evaluating chemosensitivity to guide the initial selection of chemotherapy regimens. The PDX model emerges as a pivotal tool in improving the selection and refinement of chemotherapy treatments. It aids in pinpointing sensitivity patterns, probing resistance mechanisms, and serves as a crucial platform for testing new therapeutic agents and delivery methods.

The PDX model serves as an ideal platform for investigating drug effects and mechanisms. According to Majeed et al.,^105^ TAK‐243 shows promise as an anticancer agent against SCLC, revealing insights into the role of the ubiquitin–proteasome system (UPS) in SCLC oncogenesis and progression, findings validated through PDX models. Xiao et al.^106^ reported on LXY30, highlighting its potential as a peptide for diagnosing NSCLC and delivering targeted imaging agents and cancer therapies. Further validation was conducted across various histological and genetic PDX models.

Resistance to chemotherapy in cancer cells involves complex adaptive responses that reduce their sensitivity to cytotoxic and antiproliferative effects, allowing for continued proliferation and progression within the body. This resistance significantly contributes to treatment failure in lung cancer, leading to tumor recurrence and disease advancement. Metabolic changes, including alterations in the mevalonate (MVA) pathway, play critical roles in cancer initiation and progression. The MVA pathway is vital for various cellular functions such as cholesterol production, regulation of ferroptosis, mitochondrial respiration, and synthesis of geranylgeranyl diphosphate (GGPP), which modifies small GTPases. Studies have indicated that inhibiting the MVA pathway can potentially impede cancer progression.^107^, ^108^ Chemoresistant models were established using the PDX platform through consistent administration of chemotherapy, followed by isolation and cultivation of cell lines from these drug‐resistant models. Through comprehensive drug screening and further investigation, the mechanism by which the MVA–GGPP pathway is involved in metabolic vulnerability in SCLC and represents a potentially effective therapeutic strategy for overcoming chemoresistance were revealed. LUSC ranks as the second most prevalent subtype, contributing to 20–30% of mortality from lung carcinoma. In the context of LUSC, KMT2D has emerged as the most frequently mutated epigenetic modifier, underscoring its pivotal role in driving oncogenesis through the modulation of epigenetic processes. Moreover, investigations of PDX models have shed light on the therapeutic potential of targeting KMT2D‐deficient LUSC by exploiting loss‐of‐function alterations in RTK–RAS signaling.^83^ Simvastatin plays an inhibitory role in MVA and could be a therapeutic drug for immunotherapy in CRC PDX models.^109^ The blood–brain barrier poses a physical obstacle to drug delivery for gliomas.^110^, ^111^ Qu et al.^112^ identified gambogic amide, a small molecule capable of crossing the blood–brain barrier, which demonstrated significant inhibition in PDX models and holds promise for clinical applications.

Addressing the challenge of eradicating triple‐negative breast cancer (TNBC) that is resistant to neoadjuvant chemotherapy (NACT) represents a significant and pressing clinical necessity.^113^ Echeverria et al.^114^ reported that breast tumors are capable of adopting a reversible drug‐tolerant state, which does not involve clonal selection, as a mechanism of resistance to AC therapy on the basis of treatment‐naïve TNBC PDX models and NACT‐treated TNBC patient serial biopsies. Palbociclib‐resistant breast cancer PDX models, which integrate clinical cohort data and exhibit promising potential for biomarker‐driven therapeutic selection, were employed to further investigate alternative treatment strategies.^115^

#### 3.2.3 Targeted drug investigations

In recent years, significant progress has been made in understanding the cellular signaling pathways that govern cell survival, unveiling genetic and epigenetic alterations that promote cell proliferation and tumorigenesis.^116^ However, to develop drugs that target specific genes, it is imperative to employ a model that accurately represents the heterogeneity of tumors, as the heterogeneity of tumors inhibit targeted therapy efficacy, and targeted drugs increase tumor heterogeneity.^117^ The investigation of targeted drugs necessitates the utilization of PDX models capable of monitoring authentic feedback. PDX models contain numerous gene subtypes that can be used to track distinct subclone cell responses to treatment strategies. This section presents an example of targeted drug applications in PDX models and highlights the development of some specific gene‐targeted drugs based on PDX.

Krivtsov et al.^118^ have developed a selective and orally bioavailable inhibitor that targets the menin–MLL interaction. This inhibitor effectively inhibits MLL‐fusion target genes and significantly enhances survival in MLL‐rearranged leukemia PDX models. The discovery of activating mutations in the epidermal growth factor receptor (EGFR) has identified a distinct molecular subset of NSCLC.^119^ It has been observed that most patients responding to EGFR‐targeted therapies have mutations in exons 18–21, which are part of the tyrosine kinase receptor domain.^120^ In particular, in‐frame deletions in exon 19 and the L858R mutation in exon 21 are commonly recognized as key mutations, accounting for 85% of EGFR mutations in NSCLC.^121^ Poziotinib, an oral pan‐HER inhibitor, due to its compact structure and flexible nature, has shown superior efficacy over approved EGFR TKIs in both in vitro and PDX models of EGFR Exon20ins–mutant NSCLC.^122^ In a phase II trial, objective response rates (ORRs) for poziotinib were reported at 31 and 32%, according to blinded independent review and investigator assessments, respectively. Furthermore, the trial reported a median progression‐free survival of 5.5 months.^123^ In addressing limitations of existing treatments for EGFR‐mutated NSCLC, mobocertinib (TAK‐788), a new irreversible EGFR TKI, was developed specifically to inhibit cancerous variants with activating EGFRex20ins mutations while maintaining selectivity for wild‐type EGFR.^124^ This was validated using patient‐derived models with various EGFRex20ins mutations. However, treatments with poziotinib or TAK‐788 have shown significant incidences of adverse effects related to wild‐type EGFR, such as diarrhea and rashes, limiting their clinical effectiveness.^125^ Yun et al.^126^ demonstrated that amivantamab exerts antitumor actions through multiple mechanisms in different preclinical models of EGFR Exon20ins disease. Clinical evaluation of amivantamab was further pursued based on outcomes observed in two patients with EGFR Exon20ins NSCLC treated with amivantamab. Osimertinib, an oral third‐generation irreversible EGFR TKI, selectively targets both EGFR‐TKI‐sensitizing and EGFR T790M‐resistant mutations, showing reduced activity against wild‐type EGFR. Floc'h et al.^127^ assessed the effects of osimertinib on PDX models with the G719X mutation, confirming its effectiveness and revealing its mechanism. However, resistance typically develops following osimertinib treatment, leading to disease progression. To tackle this issue, a potential therapeutic approach targeting MEK1 and AKT1/2 with costunolide has been suggested for patients resistant to osimertinib. Coadministration of osimertinib and costunolide has shown synergistic or additive effects on inhibiting tumor growth in osimertinib‐resistant cell lines and PDX models.^128^ The primary mechanism of resistance after osimertinib treatment involves a tertiary point mutation at the C797 residue of EGFR, where the cysteine in the ATP‐binding site is substituted by serine, obstructing the covalent bond formation between the mutant EGFR and Osimertinib.^128^ BBT‐176, a novel fourth‐generation tyrosine kinase inhibitor (TKI), has shown promising efficacy in patients with osimertinib‐resistant EGFR mutations in NSCLC.^129^ To combat resistance to EGFR‐targeted therapies, Marrocco et al.^130^ investigated the efficacy of early administration of combination therapies, including TKIs and antibodies, using PDX models with prevalent EGFR mutations. Zhang et al.^131^ explored a metabolic mechanism underlying resistance to molecularly targeted therapies and identified a potential therapeutic target to counter resistance to EGFR TKIs, including the third‐generation inhibitor osimertinib. The selective inhibition of AKR1B1, achieved through the use of epalrestat, an antidiabetic drug, restored the responsiveness of resistant cell lines to EGFR TKIs and delayed resistance in PDX mice with lung cancer.^131^

Human epidermal growth factor receptor 2 (HER2, ERBB2) mutations serve as oncogenic drivers in a limited fraction of NSCLCs, with occurrence rates of 2–3% among general NSCLC patients and up to 6.7% in patients negative for EGFR/ALK/ROS1.^132^, ^133^, ^134^ The most prevalent HER2 mutations consist of a 12‐base pair in‐frame insertion (p.A775_G776insYVMA) in exon 20, which activates the PI3'K–AKT and RAS–MAPK signaling pathways. Pyrotinib, an oral, irreversible pan‐HER receptor TKI, has shown efficacy against NSCLCs that harbor HER2 exon 20 mutations in PDOs and a PDX model, with clinical trials indicating promising therapeutic outcomes.^135^

Recondo et al.^136^ examined the role of Lorlatinib, a third‐generation anaplastic lymphoma kinase (ALK) inhibitor, in understanding the adaptive mechanisms underlying resistance to targeted agents through longitudinal tumor biopsy and ctDNA analysis using comprehensive molecular profiling. They also developed PDXs and cell lines to deepen this understanding.^136^ To devise more effective treatment strategies, the presence of ALK rearrangements was screened in PDX models of lung cancer, and the response to the ALK inhibitor crizotinib was evaluated. The discovery of HIP1 as a fusion partner of ALK in NSCLC marks a significant breakthrough.^137^

YES1, a cytoplasmic tyrosine kinase categorized as a member of the SRC family kinases (SFKs), has been extensively investigated. Dasatinib, the only SFK inhibitor currently approved for clinical use, is employed primarily for treating leukemia patients.^138^ Garmendia et al.^139^ indicated that YES1 status is a predictive marker for the response to dasatinib in NSCLC cells and PDX models, and it plays a vital role in the progression of NSCLC. A specific subgroup of patients likely to benefit from dasatinib treatment was identified. Redin et al.^140^ suggested that YES1 might be a novel target for SCLC treatment, with the use of dasatinib and CH6953755, potent inhibitors targeting phospho‐YES1 and YES1, leading to substantial suppression of tumor growth.

Despite the limited range of molecularly targeted drugs for SCLC, significant strides have been made in identifying and targeting driver oncogenes in lung adenocarcinoma.^141^, ^142^ These advances are attributed to a deeper understanding of the biological mechanisms of lung adenocarcinoma, particularly the identification of unique disease subtypes characterized by specific oncogenic drivers. The development of dual‐target inhibitors offers new potential for tumor treatment.^143^ Lakhani et al.^144^ introduced a novel dual‐target inhibitor that disrupts the BCL‐2/BCL‐xL complex effectively, with PDX models confirming a 30–40% reduction in tumor growth. Furthermore, combining this inhibitor with docetaxel has enhanced the efficacy in suppressing tumor growth and extending response duration. Xu et al.^145^ demonstrated the therapeutic potential of dual inhibition targeting MET and EGFR through the use of MET inhibitors (crizotinib and tepotinib) and EGFR–MET bispecific antibodies (EMB‐01 and amivantamab) in treating drug‐resistant METex14‐driven EGFR lung cancer. Schram et al.^146^ explored the effectiveness of targeting HER3 using a novel “dock and block” approach with zenocutuzumab, a HER2xHER3 bispecific drug.

ORY‐1001, a novel KDM1A inhibitor, enhances the accumulation of, facilitates blast differentiation, and exhibits significant growth inhibition in xenograft models, while also prolonging survival in a mouse PDX model of T‐cell acute leukemia.^147^

#### 3.2.4 DDS developments

The advent of nanotherapeutic strategies has instilled renewed optimism in the field of tumor treatment.^148^, ^149^, ^150^, ^151^, ^152^ The drug's limited stability, inadequate penetration of the blood–brain barrier, and suboptimal cellular uptake present substantial challenges to cancer cell absorption. Owing to the excessive expression of the ECM stroma, which constitutes a significant impediment to deep tissue penetration of chemotherapeutic agents, the inefficacy of chemotherapy in treating pancreatic ductal adenocarcinoma stems from inadequate drug accumulation within tumor tissues.^153^ Zhang et al.^154^ reported the development of a TME‐responsive nanodrug, consisting of gold nanocages coated with cell membranes, to enhance tumor tissue penetration. Nanoparticle‐based DDSs are frequently developed and utilized for targeted drug delivery. They offer significant advantages over traditional pulmonary DDSs.^154^ An encouraging strategy involves implantable drug‐loaded meshes, swiftly positioned after intraoperative assessment of tumor pathology.^155^ Specifically, porous polyglycolic acid mesh was introduced for localized delivery of paclitaxel within the tumor cavity postsurgical resection.^156^ This study introduces the initial postsurgical PDX model of NSCLC recurrence. Drug‐loaded meshes have demonstrated increased effectiveness in treating local disease, suggesting potential benefits for improving recurrence‐free survival in early‐stage NSCLC patients undergoing limited resection. A nanocatalytic sensitizer (VF/S/A@CaP) is synthesized, generating hydroxyl radicals (•OH) through the Fenton reaction, thereby inducing ferroptosis in both EGFR TKI‐resistant and EGFR TKI‐sensitive NSCLC cells.^157^

The blood–brain barrier constitutes a meticulously regulated microenvironment that governs the material interactions between blood vessels and brain tissue. Owing to their selective permeability, numerous therapeutic agents are unable to effectively traverse and reach the tumor.^158^ Studies have demonstrated that exosomes (Exos) exhibit superior abilities in traversing the blood–brain barrier (BBB) and infiltrating GBM tissue.^159^ This proficiency is likely attributable to the transferrin receptor (TfR) expressed on the surface of blood‐derived Exos, which can adsorb the endogenous transferrin present in the bloodstream, thereby facilitating a high‐affinity interaction with the TfR that is overexpressed in both the BBB and the GBM. Consequently, blood Exos were selected as combination delivery carriers for the treatment of GBM to enhance the penetration of the BBB. Drugs delivered via blood Exos effectively inhibited PDX models.

#### 3.2.5 Combination therapies

The increased use of comprehensive molecular profiling in cancer has facilitated regular detection of patients with multiple targetable alterations at diagnosis, offering opportunities for combination therapies to extend response duration and enhance targeted treatment efficacy. However, identifying optimal combinations for patients with multiple alterations remains challenging. Concurrent and specific targeting of signaling networks aims to disrupt intercellular communication and effectively address both de novo and adaptive drug resistance mechanisms. Targeting multiple nodes within a cancer‐fueling pathway using drug combinations is proposed to prevent bypass activation and improve cancer eradication. PDX provide an ideal preclinical platform to assess efficacy and potential adverse effects. A combination therapy strategy involving olaparib and temozolomide (OT) was implemented in SCLC patients, followed by PDX panel application to identify predictive molecular signatures for treatment response. Furthermore, a study showed that enhancing translesion DNA synthesis (TLS) facilitated tolerance to damage induced by OT during DNA replication. TLS inhibitors restored sensitivity to OT both in vitro and in vivo, demonstrating synergistic effects in pre‐OT treatment and post‐OT relapse PDX models from two trial participants.^160^ HER2‐expressing PDX models were subjected to combined trastuzumab deruxtecan (T‐DXd) and adavosertib therapy regimens, followed by evaluation of tumor growth through immunohistochemistry and reverse‐phase protein array analysis.^161^ ALK‐activating mutations are correlated with about 10% of neuroblastomas. The synergistic treatment strategy involving MDM2 and ALK inhibition resulted in significant tumor regression in pediatric neuroblastoma PDX models.^162^

Significantly, targeting critical paracrine signaling pathways involving cell surface receptors and their ligands can potentially directly inhibit cellular interdependence. This research focused on these pathways and demonstrated that patients with advanced‐stage NSCLC lacking targetable oncogenic mutations could benefit from a combination therapy involving cabozantinib, afatinib, plerixafor, and etoricoxib in a low‐dose protocol. PDX models treated with this regimen effectively suppressed highly therapy‐resistant adeno‐ and squamous cell carcinomas lacking targetable mutations, achieving an 81% ORR and a 100% clinical benefit rate.^163^ Tang et al.^164^ discovered that simultaneous inhibition of SHP2 and CXCR2 hindered the migration of granulocytic myeloid‐derived suppressor cells, promoting Th1 polarization and generating CD8+KLRG1+ effector T cells with increased cytotoxic potential, thereby enhancing survival outcomes across various NSCLC models. Elkrief et al.^165^ explored combining MDM2 inhibition with milademetan and MEK inhibition as a targeted therapeutic strategy in LUAD patients, particularly those reliant on MDM2 and with concurrent driver alterations.

#### 3.2.6 ADCs investigations

ADCs facilitate the targeted delivery of cytotoxic drugs to tumor cells using antibody carriers. This approach significantly enhances therapeutic efficacy by improving drug delivery and tissue penetration while minimizing toxicity. Traditional treatments for patients show limited effectiveness, with response rates typically ranging from 30 to 50% due to tumor heterogeneity, which can lead to resistance from low target expression or high intratumoral variability. The emergence of ADCs represents a promising new direction in extending benefits to a broader range of patients beyond current treatment options.

Incorporating a unique self‐immolative moiety along with the topoisomerase I inhibitor exatecan as the payload in ADCs has proven highly effective in tumors with low target antigen expression and multidrug resistance. This approach achieves lasting efficacy without increasing toxicity compared with DXd/SN‐38‐ADCs. ADCs using the T moiety–exatecan combination, such as those for EGFR‐del19/T790M/C797S triple mutation lung cancer, demonstrate prolonged antitumor effects in PDX models that accurately simulate challenging clinical scenarios. Additionally, these ADCs exhibit synergistic benefits when paired with PARP/ATR inhibitors and anti‐PD‐1 therapy. B7‐H3, a protein encoded by the CD276 gene, is overexpressed in numerous pediatric malignancies and is potentially associated with immune checkpoint treatment. Pediatric solid malignancy PDX models were utilized to confirm the efficacy of the B7‐H3 inhibitor ADC.^166^ ERBB2/HER2 is commonly expressed in many cancers, such as gallbladder cancer,^167^ breast cancer,^168^ and gastric cancer.^169^ The treatment strategies HER2 ADC–T‐DXd and trastuzumab emtansine (T‐DM1) were investigated in desmoplastic small round cell tumor (DSRCT) PDX models and showed a strong response. Although HER2–ADC treatment has been reported to be effective for treating breast cancer,^170^ NSCLC,^171^ and urothelial carcinoma.^172^ The therapeutic potential of HER2–ADC in PDX models, such as gallbladder cancer, which poses a significant threat to patients and requires capturing its heterogeneity to investigate new treatment strategies, warrants further exploration.

#### 3.2.7 In radiotherapies

The issue of cancer resistance to radiotherapy remains a significant challenge in clinical oncology. A key factor contributing to tumor radioresistance is the presence of cancer stem cells (CSCs), known for their capacity to self‐renew and generate diverse cancer cell types. Studies indicate a direct correlation between a higher proportion of CSCs within tumors and increased resistance to radiation therapy. CSCs can repopulate and recover from radiation‐induced damage between treatment sessions, which enhances resistance to fractionated radiotherapy. The survival of CSCs postradiation treatment is frequently associated with tumor recurrence.

Choi et al.^173^ investigated the characteristics of sphere cells formed from ionizing radiation (IR)‐treated PDX tumors, opening new avenues for targeted therapeutic strategies against radioresistance. They identified significant upregulation of SERPIB4 and CCL2 in sphere cells derived from IR‐treated tumors compared with those from non‐IR‐treated tumors, highlighting their roles in cancer‐related immune responses. While external beam radiation is effective in localized treatment, its limitation lies in addressing the widespread and metastatic nature of lung cancer. Systemic radioimmunotherapy, utilizing a monoclonal antibody specific to DLL3 expressed exclusively on cancer cells, offers potential by delivering targeted radiation to disease sites. The low toxicity profile of [177Lu]Lu–DTPA–CHX‐A–SC16, validated in PDX models targeting DLL3, positions it as a promising candidate for clinical application in treating lung cancer.^174^

### 3.3 Integration of the PDX model and omics

The utilization of PDX models provides a highly relevant system for investigating cell–cell communication, while omics analysis offers valuable insights into the interactions that drive tumor growth and progression by identifying the specific mutation information originating from cells within the tumor microenvironment. These findings form the basis for further clarifying the speculative interactions and validating the molecules involved in tumor‐associated biological processes.

#### 3.3.1 PDX model combined with multiomics technology

The utilization of PDXs, combined with the incorporation of whole‐exome sequencing (WES) and RNA‐seq analysis, has enabled the enhancement of assay sensitivity and the application of stringent criteria for identifying cancer‐associated genes.^175^, ^176^ RNA‐seq and whole‐exome analysis of the prostate cancer PDX tissue substantiated the integral role of PDX models as a preclinical platform for monitoring drug response.^177^

Acute myeloid leukemia PDX's heterogeneity were investigated through transcriptome and exome sequencing.^178^ The proteomic and transcriptomic analyses' findings were verified using PDX models. Ramkumar et al.^179^ conducted these analyses on cell lines and tumor tissues, pinpointing BCL2 as pivotal in SCLC cells' inherent resistance to AURKB inhibitors. Consequently, a negative correlation was observed between the expression of BCL2 and the in vivo response to AZD2811 (AURKB inhibitor) in PDX models derived from either CTCs or biopsies obtained after relapse to initial platinum therapy.^179^ To identify potential targets, deep proteome profiling and CRISPR‐Cas9 screening were conducted on acute leukemia PDX models.^177^

To explore the implications of low‐frequency variants on drug resistance, mice hosting PDXs of lung cancer underwent treatment with G12C inhibitor therapy, followed by subsequent DNA sequence analysis. Single‐cell DNA sequencing, targeted bulk exome sequencing, and WES were employed on G12Ci‐resistant cell lines, revealing a diverse resistance pattern characterized by the emergence of multiple subclonal events after G12 Ci treatment. Targeting ERK signaling intermediates either genetically or pharmacologically amplified the antiproliferative effects of G12 Ci treatment in models exhibiting acquired RAS or BRAF mutations.^180^

Genomic, epigenomic, transcriptomic, and proteomic analyses were conducted on microdissected components of mixed histology tumors, as well as pre‐ and posttransformation tumors, alongside reference nontransformed samples of LUAD and LUSC. Quintanal‐Villalonga et al.,^181^ utilizing PDX validation, provided a comprehensive understanding of LUSC transdifferentiation, uncovering key drivers and potential therapeutic targets to effectively manage or prevent lineage plasticity. Integration of multiomics data in PDX of neuroendocrine prostate cancer revealed the presence of shared clinicopathological characteristics.^182^

Sun et al.^183^ conducted a thorough genomic characterization of PDX models by analyzing WES and RNA‐seq data from both human samples and PDX samples obtained from the PDX metadata bioinformatic library. This study represents the largest‐scale investigation to date, including analyses of driver mutations, gene fusions, and copy number variations (CNVs).^183^

A broad cohort of 137 NSCLC PDX models was established and thoroughly characterized, involving analysis of genes, DNA methylation, gene CNV, exome mutations, pY‐proteomes, and proteomes.^184^ Two proteotypes were identified for LUSC and three for LUAD, each showing correlations with patient survival. The data analysis suggests that genomic features remain largely consistent throughout the engraftment process. Furthermore, the proteomic platform demonstrated a high level of consistency and accuracy, with minimal proteome remodeling observed during the metastasis and serial passaging of PDX tumors. Analysis of subprototype data unveiled distinctly different stromal proteomes, suggesting that proteotype‐specific gene signatures may attract unique stromal cells to the microenvironment. This observation also suggested that PDX models could serve as convenient and ideal platforms for further experimentation with CAFs. The data highlighted multiple candidate targets linked to proteotypes, presenting promising opportunities for addressing NSCLC.

Lissa et al.^185^ integrated transcriptomic and genomic data to explore the variability in neuroendocrine transcriptional states within metastatic SCLC tumors and compared these findings with PDX models. They identified a unique subtype of neuroendocrine differentiation in SCLC and observed enrichment of tumor cells with this subtype, indicating clonal selection within the PDX models. Their combined genomic and transcriptomic analyses provided insights into mutational profiles, gene expression patterns, and subtype classifications specific to SCLC PDX models.^186^ This comprehensive approach serves as a valuable resource for understanding SCLC biology and guiding the development of clinical treatment strategies. Quintanal‐Villalonga et al.^187^ utilized a multiomics approach to demonstrate that pharmacological inhibition of the PI3K/AKT pathway reduced tumor growth and suppressed neuroendocrine transformation in a PDX model harboring EGFR mutations. They conducted a thorough proteogenomic analysis of SCLC samples obtained from 112 patients who underwent surgical resection of lung tumors and adjacent tissues. Integrated multiomics analysis identified four distinct subtypes with unique therapeutic vulnerabilities, which were validated through drug testing in PDX models, underscoring the predictive utility of multiomics subtyping.^85^

#### 3.3.2 PDX model combined with single‐cell analysis

Bulk tissue networks mainly represent the combined activities of cell populations, focusing on common pathways or functions rather than individual cell behaviors. While such bulk analyses provide a broad overview of interaction networks, they offer a limited understanding of direct interactions. Integrating single‐cell multiomics approaches can enhance interpretability by inferring directionality. The rise of high‐throughput single‐cell omics technologies has significantly broadened the possibilities for exploring potential targets and treatment strategies in lung cancer.^176^ The integrated applications of single‐cell analysis and PDX models were categorized into two sections: validation analysis on PDX and investigation analysis from PDX tissues. However, sometimes novel single‐cell analysis method was tested on the PDX models.^188^ A novel single‐cell sequencing of transposases (scGET‐seq),^188^ and a PROSPERO assay was introduced by Panovska et al.^189^ were both evaluated on PDX models. The crucial key gene of RUNX1 was identified through screening pancreatic cancer (PC) gemcitabine‐based chemo‐resistant single‐cell genes. Subsequently, the validation of PDX confirmed RUNX1 as a predictive biomarker for overcoming gemcitabine resistance PC.^190^ Six renal cell carcinoma PDX models were performed on single‐nucleus RNA sequencing analysis to confirm the feasibility of the combination the molecular guided strategy.^191^ Chen et al.^192^ employed single‐cell analysis to investigate osimertinib‐resistant NSCLC, validating in PDX models the effectiveness of combination therapy targeting multiple driver alterations simultaneously, which led to improved therapeutic outcomes. Moghal et al.^193^ utilized PDX models and single‐cell RNA sequencing to identify a rare subpopulation of cells, constituting about 4% of the total population, with transcriptomic similarities to drug‐tolerant persister (DTP) cells. These cells displayed intermediate activity in pathways upregulated in DTPs. The study hypothesized that TKIs activate dormant tumor‐propagating cells, transforming the predominant cancer‐associated fibroblast population into a state that enhances the survival of these cells. DTP PDX models were established by administering daily doses of 50 mg/kg erlotinib or 25 mg/kg osimertinib via oral gavage, excluding weekends, over a 30‐day period. Integrating single‐cell analysis and PDX models highlighted the DTP and posttreatment CAF pathways, focusing particularly on the NF‐kB and IL‐6/JAK/STAT3 pathways identified in this research. These pathways warrant further investigation as potential targets for therapeutic strategies aimed at reducing recurrence rates in patients.

Intratumoral cell heterogeneity is associated with clinical outcomes in cancer patients. The most direct approach for investigating tumor heterogeneity involves the implementation of single‐cell sequencing techniques. However, owing to challenges in obtaining continuous tumor samples during different drug administration periods or the requirement for additional processing of acquired tumor samples, PDX models present an exceptional platform that fulfills researchers' needs for studying the status of tumor models. Kim et al.^194^ performed single‐cell analysis on PDX tumor tissue and identified a candidate subgroup associated with drug resistance. Two opposite drug response breast cancer PDX tumor tissues were directly investigated through the single‐cell analysis.^195^ Novel intermediate transcriptional states cell subtype were confirmed by the scRNA‐seq data of TNBC.^196^ The resistance of PDX models to chemotherapy was investigated using single‐cell analysis.^197^ This analysis revealed an overall increase in intratumor heterogeneity in both chemosensitive and chemoresistant PDX models, showing diverse expression patterns of potential therapeutic targets and resistance pathways, including EMT, among different cellular subpopulations following treatment resistance. The study identified that treatment resistance in SCLC is associated with the presence of various subpopulations exhibiting diverse gene expression profiles, which collectively contribute to multiple simultaneous resistance mechanisms. However, the study did not explore potential links between immune cell presence and chemotherapy effectiveness.

The application of single‐cell analysis has significantly increased our understanding of PDX. This technique can reveal the transcriptional characteristics of diverse cell types within the tumor microenvironment, including drug‐resistant cells and stromal cells, thereby offering a fresh perspective for comprehending the intricacy and heterogeneity of tumors. Moreover, integrating the PDX model with single‐cell sequencing also facilitates functional testing of drug sensitivity, providing valuable insights for developing personalized treatment strategies.

### 3.4 Clinical trial

The limited success rate of drugs in human clinical trials, primarily attributed to inadequate efficacy, necessitates the exploration of alternative approaches, such as sufficiently powered trials employing PDX in murine models to assess therapeutic activity effectively across diverse tumor types. The latest utilization of PDX models has been in “coclinical trials,” also referred to as “avatar” or “mirror” models, which are developed from specimens obtained from patients enrolled in clinical trials. These pre‐clinical studies are conducted concurrently and in real time with human trials. Coclinical trials represent a burgeoning field of research wherein a clinical trial is conducted in tandem with a corresponding preclinical trial, either subtype‐matched or patient specific, to provide insights and inform the clinical trial.

The emergence of PDXs as coclinical platforms is driven primarily by the recognition that established PDX models faithfully recapitulate patients' tumors in terms of histomorphology, gene expression profiles, and gene copy number alterations while also demonstrating their ability to accurately predict therapeutic response in patients, particularly when clinically relevant drug dosages are employed. Evrard et al.^198^ conducted a study involving four blinded PDX development and trial centers to confirm the robustness of PDX models for drug response and experimental data assessment. Moreover, PDX were used in a clinical trial for validation. A cohort of 50 patients diagnosed with World Health Organization (WHO)‐defined chronic myelomonocytic leukemia was enrolled in an open‐label, multi‐institutional phase 1/2 clinical trial, in which ruxolitinib administered twice daily was evaluated during phase 2. Concurrently, 49 PDX originating from 13 study participants were established and subsequently randomized to receive either ruxolitinib or a vehicle control.^199^ Five PDX tumors were evaluated for drug efficacy in a coclinical trials for the prediction of potential biomarkers.^200^ The PDX model was employed within the framework of a “coclinical trial” approach. In this context, the PDX model is derived from a patient enrolled in a clinical trial and subjected to identical experimental agents to replicate the clinical response. This enables simultaneous assessment of drug response in both the patient and the mouse model, thereby providing an invaluable platform for real‐time investigations of resistance mechanisms, predictive biomarkers, and innovative combination strategies.

Phase I clinical trials offer a distinctive opportunity that can enhance the understanding of novel anticancer therapies, thereby highlighting the guidance of clinical development. DiPeri et al.^201^ demonstrated the feasibility of predicting drug sensitivity in clinical trials through PDX models. PDX models represent an ideal investigative platform involving the use of the most patient‐relevant tools for cancer research because of their accurate reflection of the molecular profiles of patient tumors. The growth inhibitory effects of zanidatamab were observed in a breast cancer PDX model, which demonstrated a consistent drug response among both patients with a partial response and patients with progressive disease. The similar antitumor efficacy observed in PDXs and actual patients strengthens the validity of employing PDXs as a clinically relevant model in both basic and translational research. The amplification of MET is proposed as a potential mechanism underlying acquired resistance to zanidatamab (HER2 inhibitor). Consequently, the use of MET inhibitors, both as monotherapies and in combination with other treatments, is being explored as a viable therapeutic strategy. The immune drug response to the treatment in this clinical trial, however, was not detected. The use of humanized mice should be considered for further experiments. Mice transplanted with GBM cells were incorporated into a multimodal platform for the comprehensive analysis of drug efficacy and response, offering high spatial resolution for clinical testing and providing a biomarker signature indicative of drug effectiveness.^202^ Early in 2015, Guo et al.^55^ conducted in vivo drug screenings utilizing a 1 × 1 × 1 (one animal per model per treatment) PDX clinical trial to evaluate population‐level responses to 62 treatments across six cancer indications. They posited that this experimental paradigm could provide predictive insights into therapeutic responses. In 2016, the Public Repository of Xenografts (PRoXe; www.proxe.org), which is an extensive, publicly accessible repository of well‐characterized leukemia and lymphoma PDXs, conducted pilot studies to facilitate direct comparisons between drug‐treated and control groups within the same cancer model.^203^ Numerous clinical trials have necessitated the sacrifice of a substantial number of mice; however, it is noteworthy that in the same year, Murphy et al.^204^ evaluated data from 67 compounds combined with PDX models for drug response prediction to address this issue. In 2021, utilizing a 1 × 1 × 1 experimental design, Risbridger et al.^205^ efficiently identified 59 PDX tumors that exhibited exceptional responses to combination therapies. To manage the distribution of PDXs, the Melbourne Urological Research Alliance was established. Yao et al.^206^ conducted 49 HNSCC PDX models and randomized “Phase II‐like clinical trials,” which identified potential biomarkers related to intrinsic resistance to cetuximab.

ADCs, such as T‐DXd, are markedly revolutionizing the therapeutic landscape for both localized and metastatic solid tumors. T‐DXd consists of the trastuzumab linked to deruxtecan, which includes a cleavable tetrapeptide‐based linker and a topoisomerase I inhibitor. Kabraji et al.^207^ demonstrated that T‐DXd effectively reduces tumor growth and extends survival in PDX models of HER2‐positive, HER2‐low, and T‐DM1–resistant active breast cancer brain metastases (BCBM). T‐DXd demonstrated a central nervous system ORR of 73% (11 out of 15) in patients with BCBMs who had undergone extensive prior treatment. Among the 10 participants with either untreated or progressive BCBMs, seven exhibited a partial response, whereas three maintained stable disease. Moreover, apoptosis may serve as one mechanism through which T‐DXd induces tumor regression and extends survival in animal models.

## 4 CONCLUSION AND PROSPECTS

The comprehensive investigation of the PDX model presents significant prospects for the advancement of pharmaceutical and clinical medicine. Due to its ability to preserve the heterogeneity and pathological characteristics of patients, PDX models can faithfully replicate patients' true drug responses, the mutational processes underlying drug resistance pathways, subpopulation dynamics, tumor absorption kinetics, and intercellular communication within the tumor microenvironment. Consequently, PDX models serve as an indispensable platform for future drug research and development as well as personalized precision medicine, effectively bridging the gap between clinical and basic research. Maximizing the potential of PDX platforms is crucial for unlocking novel cancer therapeutics in the future and remains a pressing challenge that needs further attention.

While PDX models offer promising advancements in cancer research, it is essential to acknowledge and tackle their significant limitations to make them more accessible for translational and preclinical studies. Using PDX models requires substantial financial investment and access to well‐equipped laboratory facilities. Key challenges that need attention and standardization to promote wider adoption include the time needed to generate PDX tumor tissues, the substitution of human stroma with murine stroma, and the current lack of immune system assessment.

PDX models are not suitable for early‐stage cancers due to their low success rate and the prolonged tumor latency of 4–6 months, which is a significant limitation for personalized medicine. When creating metastatic models, both heterotopic and orthotopic grafting methods, as well as injecting patient‐derived tumor cells into mice via the tail vein, each have their inherent advantages and limitations. Spontaneous metastasis in grafted tumors occurs slowly with a low rate, whereas injecting tumor cells results in a higher metastatic rate but presents challenges related to unrealistic heterogeneity, often leading to most cells being confined to the lungs rather than spreading to other organs.

**FIGURE 5:**
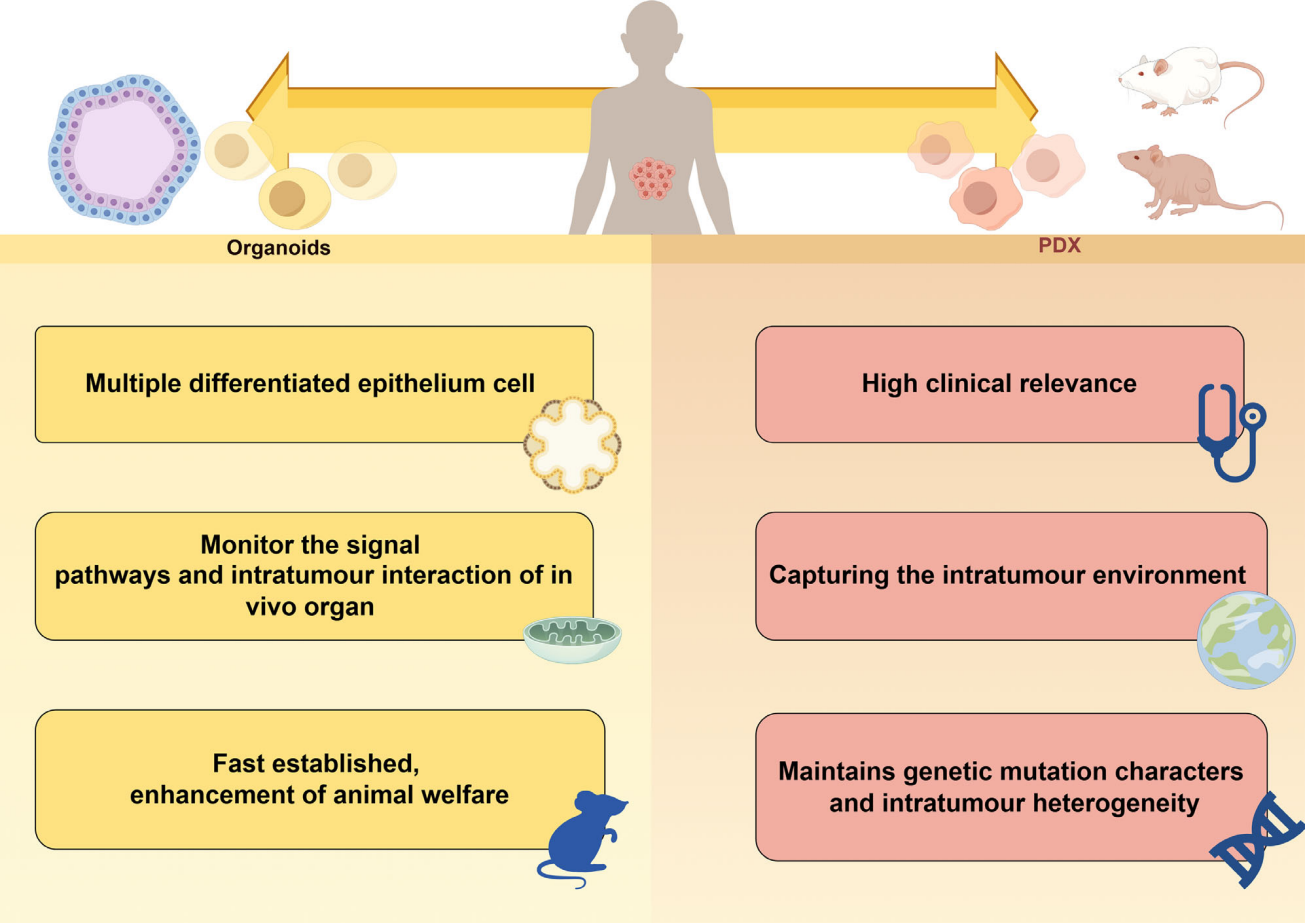
The comparisons of advantages of PDO and PDX. The patients’ tumor can be used to establish PDX and PDO simultaneously. And PDO and PDX have their own advantages. PDOs, serving as in vitro models, can be employed as a rapid and animal welfare‐friendly approach to monitor patients’ response to tumor drugs and investigate underlying mechanisms. PDXs are considered highly clinically relevant preclinical platforms and in vivo models that capture the intratumoral environment while preserving tumor heterogeneity.

Reconstructing autologous tumor stroma and a functional human immune system are critical for preserving tumor heterogeneity in PDX models and enhancing their relevance in translational medicine. The complexity of generating PDXs may pose challenges for long‐term studies. As research advances, obtaining immune system and stromal cells directly from clinical patients may become increasingly difficult. The emergence of PDOs offers an additional avenue for precision medicine and the evaluation of clinical drug efficacy. PDOs can effectively recapitulate the pathological and genetic characteristics of patients’ tumors, but their rapid establishment, cost effectiveness, ability to reduce the input of animal models to increase the number of animals appendages and minimal culture requirements make them promising alternatives to animal models. However, as an in vitro model, PDOs alone cannot fully bridge the gap with in vivo models unless complemented by PDOX models. The integration of PDOs and PDXs as complementary references represents an ideal approach for future drug screening and precision medicine endeavors. Achieving concordant results between in vivo and in vitro human model platforms holds greater credibility and accuracy for medical research. The comparisons of advantages of PDO and PDX were showed in Figure 5.

The PDX model is renowned for its ability to accurately replicate tumor heterogeneity and faithfully mirror clinical responses to drugs, making it an excellent tool for preclinical drug screening. Looking ahead, patient‐derived models such as PDXs hold promise for accelerating the discovery of new drugs and enhancing our understanding of disease progression and resistance to therapies. Extensive research in PDX models aims to unravel intricate relationships between potential biomarkers and tumor development, with a focus on refining cancer treatment strategies. Screening and validating combination therapies and novel drugs in PDXs provide reliable preclinical data for translational research. Moreover, integrating omics technologies with PDX models enables ongoing monitoring of tumor heterogeneity and specific characteristics like clonal metastasis potential. Expanding the application of PDX models, particularly through the incorporation of stromal or immune cells, represents a significant advancement. Ultimately, PDX offer a pathway to advancing personalized treatment approaches, marking a substantial stride forward in translational medicine.

## AUTHOR CONTRIBUTIONS

Y. L. and M.‐Y. L. conceived the review. A. G. drafted and wrote the manuscript. J. L. participated in part of the writing, M.‐Y. L. reviewed and edited the manuscript. All authors approved the final version of the manuscript.

## CONFLICT OF INTEREST STATEMENT

The authors declare that they have no conflict of interest.

## ETHICS STATEMENT

Not applicable.

## Data Availability

Not applicable.
